# The SarcoEndoplasmic Reticulum Calcium ATPase (SERCA) pump: a potential target for intervention in aging and skeletal muscle pathologies

**DOI:** 10.1186/s13395-021-00280-7

**Published:** 2021-11-12

**Authors:** Hongyang Xu, Holly Van Remmen

**Affiliations:** 1 Aging & Metabolism Research Program, Oklahoma Medical Research Foundation, Oklahoma City, OK 73104 USA; 2 Oklahoma City VA Medical Center, Oklahoma City, OK USA; 3 Department of Physiology, OUHSC, Oklahoma City, OK USA

**Keywords:** Skeletal muscle, SERCA, Calcium homeostasis, Aging, Neuromuscular disorder, Oxidative stress

## Abstract

As a key regulator of cellular calcium homeostasis, the Sarcoendoplasmic Reticulum Calcium ATPase (SERCA) pump acts to transport calcium ions from the cytosol back to the sarcoplasmic reticulum (SR) following muscle contraction. SERCA function is closely associated with muscle health and function, and SERCA activity is susceptible to muscle pathogenesis. For example, it has been well reported that pathological conditions associated with aging, neurodegeneration, and muscular dystrophy (MD) significantly depress SERCA function with the potential to impair intracellular calcium homeostasis and further contribute to muscle atrophy and weakness. As a result, targeting SERCA activity has attracted attention as a therapeutical method for the treatment of muscle pathologies. The interventions include activation of SERCA activity and genetic overexpression of SERCA. This review will focus on SERCA function and regulation mechanisms and describe how those mechanisms are affected under muscle pathological conditions including elevated oxidative stress induced by aging, muscle disease, or neuromuscular disorders. We also discuss the current progress and therapeutic approaches to targeting SERCA in vivo.

## The role of SERCA in skeletal muscle metabolism

Skeletal muscle is the largest organ in the body, contributing close to 40% of total body mass. It plays a major role in metabolism and key physiological and biochemical processes in addition to its critical functions of force generation and movement [25]. Contractile force generation is a process mediated by calcium ions resulting in the activation of interaction between myosin and actin filaments [30]. As a result, the regulation of calcium homeostasis is critical to proper maintenance of muscle function. A key regulator of cellular calcium homeostasis is the Sarcoendoplasmic Reticulum Calcium ATPase (SERCA) pump which acts to transport calcium ions from the cytosol back to the sarcoplasmic reticulum (SR) following muscle contraction. The SERCA protein is localized on the SR membrane and has been reported to be the most abundant protein in the SR [6].

**Fig. 1:**
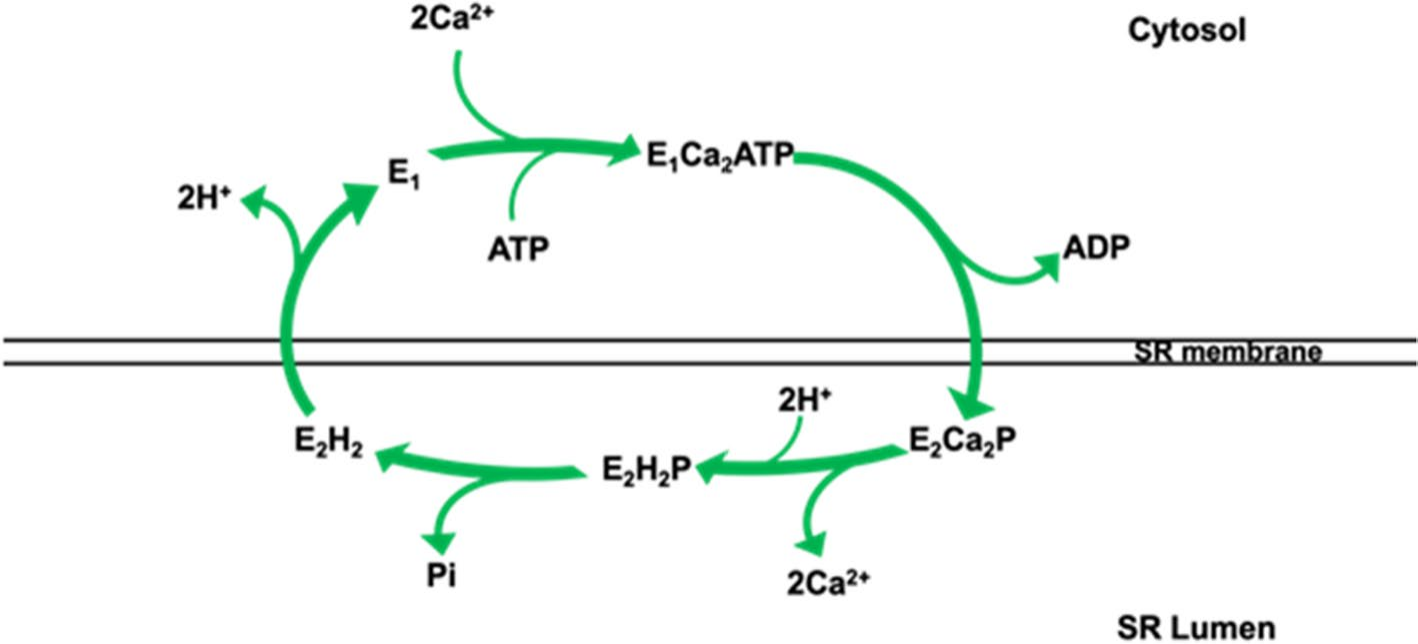
Schematic diagram of the kinetic cycle of SERCA pump. During the transportation of Ca^2+^ ions, ATP binding is coupled with the change of conformational states of SERCA from E_1_ to E_2_. These two states have different affinity to Ca^2+^ ions, with E_1_ being highly affinitive and E_2_ being low. Each time, there are two Ca^2+^ ions and an ATP molecule binding to E_1_ state (E_1_Ca_2_ATP), then after the hydrolysis of ATP the state of SERCA is changed to E_2_ (E_2_Ca_2_P) with the release of ADP. The release of Ca^2+^ ions into the SR lumen requires the exchange of luminal protons on E_2_ state (E_2_H_2_P). The final step is dephosphorylation and dehydrogenation to return the enzyme to the ground state, E_1_, which then is able to initiate a new transport cycle. Figure reproduced based on Periasamy et al. 2007 [51]

**Fig. 2:**
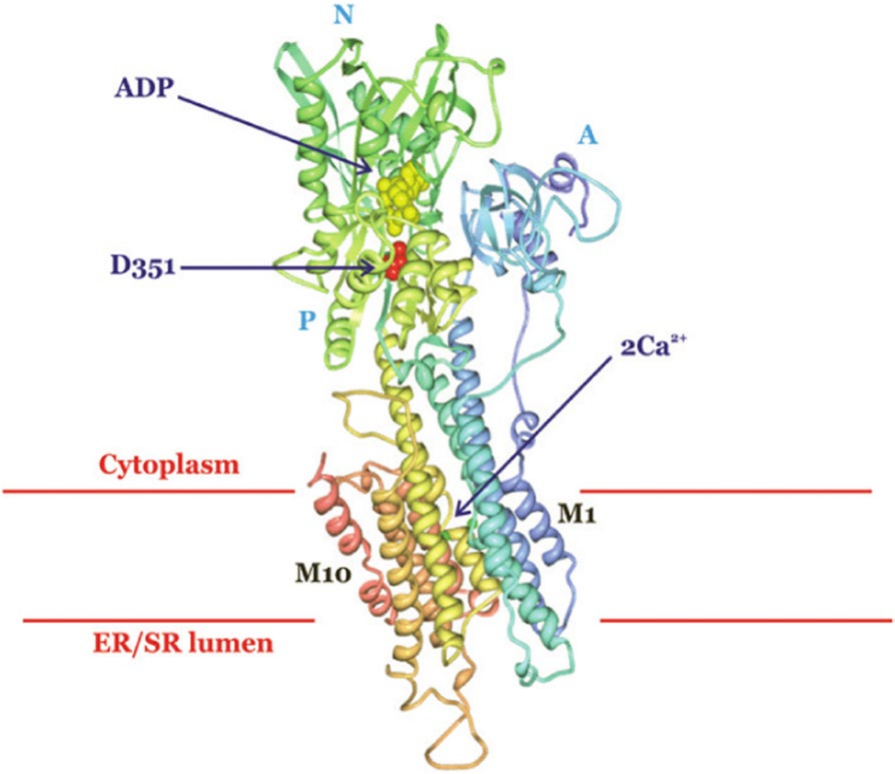
Schematic diagram of the structure of sarcoendoplasmic reticulum calcium ATPase (SERCA). The 3-dimensional crystal structure shows a SERCA pump in the ADP- and Ca^2+^-bound state. D351 (in red) is the residue phosphorylated during the movement of Ca^2+^ ions into the ER or SR. The three cytoplasmic domains, phosphorylation (P), nucleotide binding (N), and actuator (A) are labeled. ADP is shown in yellow and Ca^2+^ ions in green. Figure adapted from [69]

The SERCA pumps belong to the family of P-type ATPases that includes a series of membrane-bound ATPases, such as plasma membrane Ca^2+^ ATPase (PMCA), Na^+^/K^+^ ATPase, and H^+^/K^+^ ATPase [64]. A common feature of these P-type ATPases is to transport metal ions against the gradient across the SR membrane coupled with the hydrolysis from ATP to ADP [47, 48]. As illustrated in Fig. 1, the primary function of SERCA is the uptake of cytosolic Ca^2+^ back into SR lumen using energy derived from the hydrolysis of ATP. This allows the maintenance of the cytosolic Ca^2+^ concentration at low levels between 50 and 100 nM [5]. Figure 2 shows the structure of the SERCA pump as reported by Watson in 2015 [69], revealing a globular lobe that protrudes into the cytosol connecting with the SR membrane through a stalk that has only a minor extension into the lumen of the SR [5].

During excitation-contraction (E-C) coupling events associated with muscle force generation, Ca^2+^ ions are released through the ryanodine receptor (RyR) channels in the SR membrane increasing the cytosolic [Ca^2+^] to 1–2 μM for a few milliseconds. This high concentration of Ca^2+^ ions facilitates the interaction of calcium with troponin to trigger the sequence of events leading to force production [33]. However, prolonged high cytosolic [Ca^2+^] can be detrimental to cellular homeostasis, stimulating calcium signaling pathways and leading to activation of proteases such as calpains [44] and matrix metalloproteinases (MMPs) that can degrade cellular components [58]. Moreover, calcium ions can directly control contractile function in muscles, especially in cardiac muscle, where the intracellular calcium release from the SR is triggered by a small influx of calcium, which is termed as calcium-induced calcium release (CICR). Elevated dysregulated calcium concentration can directly contribute to adverse cardiac remodeling and disruption of systolic and diastolic function [36]. Thus, dysfunctional SERCA pumps could contribute to high cytosolic calcium, limiting not only muscle function, but also causing impairments in cellular metabolism and function.

The primary goal of this review is to provide an overview of the impact of pathological conditions such as high oxidative stress induced by aging, muscle disease or neuromuscular disorders on SERCA function, and the potential therapeutic approaches via targeting SERCA.

## SERCA pump isoforms and their regulation

The SERCA pump family is encoded by three different genes, SERCA 1, SERCA 2, and SERCA 3, and seven different isoforms are expressed from these genes, SERCA 1a/1b, SERCA 2a/2b, and SERCA 3a/3b/3c [51] (Table 1). SERCA 1 is a skeletal muscle specific isoform expressed predominantly in fast-twitch muscles, with 1a and 1b being adult and neonatal forms respectively. SERCA 2a is primarily expressed in slow-twitch skeletal muscle and cardiac muscle, while SERCA 2b is a ubiquitous isoform appearing in all cell types at a low abundance. SERCA 3 is very rare in muscle tissues but is universally expressed in non-muscle tissues (i.e., neuronal cells and epithelial cells) at a very low level [52]. The structure of SERCA pumps is highly conserved despite the fact that they are encoded from several different genes, with SERCA 1 showing 84% similarity to SERCA 2a and 75% to SERCA 3. Because of their similar structures, it has been predicted that all SERCA isoforms might have similar native transmembrane arrangements and tertiary conformations, and hence, their sensitivity to Ca^2+^ and enzyme activity should be equivalent [38]. In particular, the muscle isoforms SERCA 1 and SERCA 2a have enzymatic properties that are almost identical [51].

**Table 1:** SERCA isoforms and its distribution in mammalian tissues

Cell type	SERCA isoforms
Skeletal muscle	
*Fast twitch*	SERCA 1a, 1b*, 2a, 2b (*fetal only)
*Slow twitch*	SERCA 2a, 2b
Cardiac muscle	SERCA 2a, 2b
Non-muscle cells	SERCA 2b, 3a, 3b, 3c

The activity of the SERCA pumps is regulated by a series of small molecular weight proteins present in muscle, including phospholamban (PLN), sarcolipin (SLN), the dwarf open reading frame (DWORF), and the recently identified myoregulin (MLN) [4, 39, 46, 61]. All these regulatory proteins are expressed differentially in muscle; however, PLN, SLN, and the newly discovered MLN share a highly conserved hydrophobic motif in the trans-membrane region which is rich in leucine residues, providing an interaction surface for binding to SERCA. PLN is found to be expressed in all muscle types, but primarily interacts with the SERCA 2a isoform in cardiac and slow-twitch muscles. Compared to PLN, SLN can regulate both SERCA 1 and 2 isoforms [51]. Both PLN and SLN are found to be less effective on regulating SERCA 3 isoform, whereas in contrast, DWORF is found to be able to bind and regulate all three isoforms of SERCA, including SERCA 3 [46]. The physiological role of PLN in cardiac muscle and SLN in skeletal muscle has been well established with similar inhibitory effects on SERCA activity, but through different mechanisms. PLN inhibits SERCA activity by reducing the SERCA pumps affinity to Ca^2+^ decreasing ATPase activity, while in the case of SLN, the ATP activity is not affected, but the maximum calcium uptake rate is reduced, suggesting that there is a Ca^2+^ uncoupling function of SLN to SERCA [60]. Interestingly, SLN also plays an important role in regulating muscle thermogenesis. SLN mediates the uncoupling of Ca^2+^ ions and SERCA, which allows SERCA to execute a futile cycle of hydrolysis of ATP to ADP which generates heat without pumping Ca^2+^ ions, thus contributing to temperature homeostasis of the body, another critical function of muscle beyond contraction [49]. MLN function is still an emerging area of research, and recent studies suggest that MLN exerts an inhibitory regulation on SERCA activity; indeed, the genetic ablation of MLN was able to improve the SERCA function and enhance exercise performance [46]. In contrast, DWORF functions differently from the others in that it is an activator of SERCA. It has been reported that in cardiac muscle, genetic manipulation replacing PLN with DWORF results in a dramatic increase in SERCA activity and the muscle contractility is also improved [46].

## Age related in changes in SERCA activity

Aging is a progressive process that is marked by a gradual compromise in biochemical and physiological capabilities resulting in reduced organ function. As a result, aging is the major risk factor for the development of cancer, neurodegenerative disorders, cardiovascular diseases, and neuromuscular myopathy [67]. A key cellular process associated with aging is oxidative stress, especially in skeletal muscle where a higher ROS production rate is associated with aged muscle [40, 55]. In particular, oxidation and nitration of cellular protein has been suggested to be an underlying causal factor in the progressive loss of cellular functions [63]. Oxidative stress can exert a feedforward effect in which elevated oxidation of cellular components can further exacerbate the cellular oxidative stress environment and impair protein targets that are sensitive to oxidative inactivation. Unchecked, these protein modifications can promote mutations of nuclear and mitochondrial DNA (mtDNA), downregulating energy metabolism, generating more ROS and other harmful molecules, and further accumulating an even higher oxidative stress in aging cells [17, 63].

### Oxidative stress impacts SERCA function and has consequent effects on skeletal muscle

**Fig. 3:**
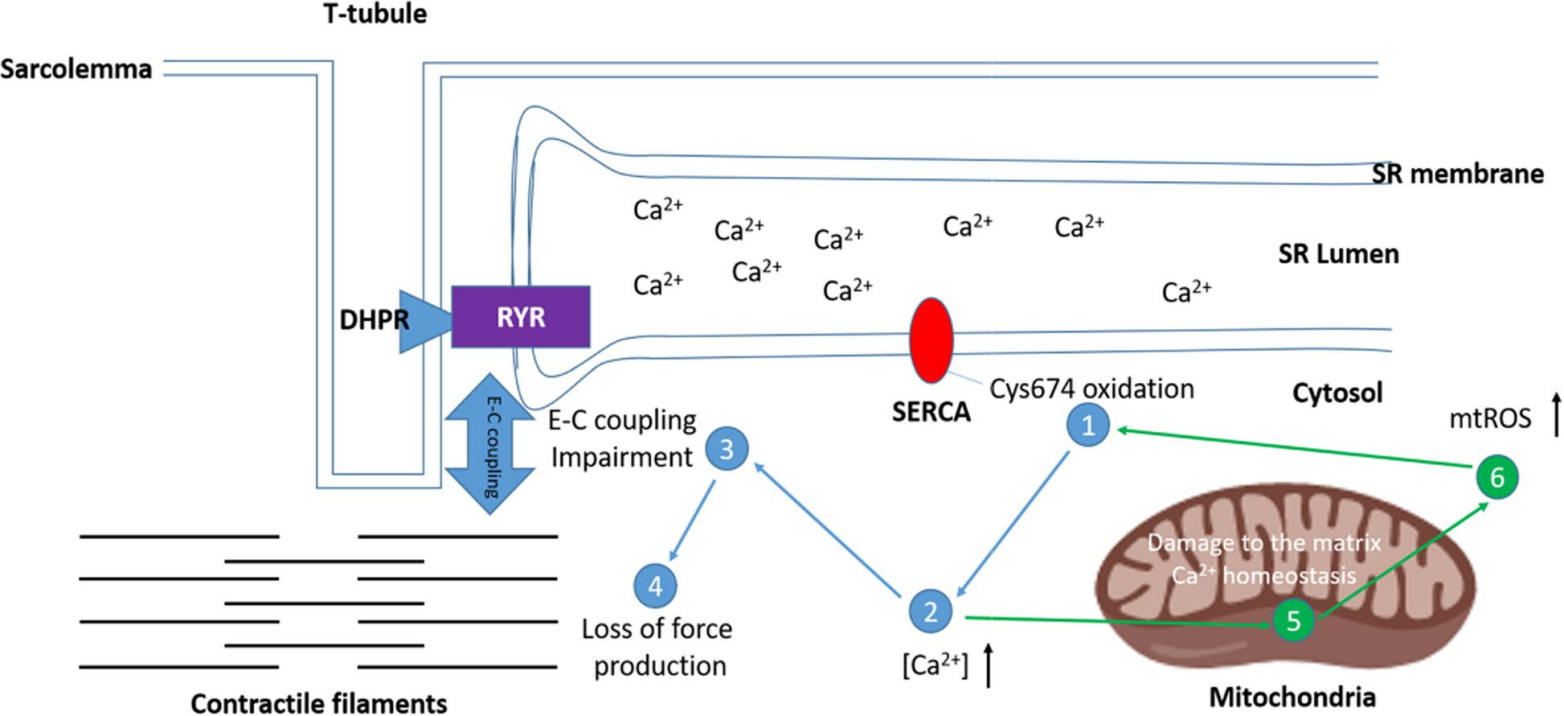
Schematic diagram of the response to oxidative damage of the SERCA pump. Higher cytosolic oxidative stress irreversibly oxidizes the Cys674 residue of SERCA (step (1)) resulting in a reduced SERCA activity. The reduced SERCA activity increases the cytosolic Ca^2+^ concentration (step (2)). The high cytosolic [Ca^2+^] is detrimental to E-C coupling (step (3)) and eventually induces the decline in muscle force (step (4)). High cytosolic [Ca^2+^] also results in the impairment of mitochondrial Ca^2+^ homeostasis (step (5)), which leads to increased generation and release of mtROS to the cytosol (step (6)), and the elevated mtROS will trigger additional oxidation of SERCA (step (1)) exacerbating the damage elicited by oxidative stress

Commonly, oxidative stress is defined as the imbalance between the generation of pro-oxidants and antioxidant response, leading to an accumulation of oxidized molecules [9]. As mentioned above, aging has been associated with an increased oxidative stress, and in turn, the SERCA pump has been shown to be susceptible and sensitive to oxidative modifications. The first report that a reduced SERCA activity occurs in biological aging demonstrated a selective oxidation on cysteine residues of SERCA proteins [62]. More specifically, cysteine 674 was found to have an irreversible oxidation mediated by elevated hydrogen peroxide treatment. In cardiac muscle, oxidation on Cys674 dramatically reduced the SERCA activity and, importantly, impaired the cardiac myocyte relaxation in aged mouse heart. Genetic modification to replace the Cys674 with serine (Cys674Ser) resulted in a smaller degree of SERCA inactivation in response to oxidation by ROS [56]. In addition to cardiac muscle, Dremina et al. found cysteine residues in SERCA in skeletal muscle that can be modified by oxidation, including Cys674, Cys675 in SERCA 1, and Cys674 in SERCA 2 [12]. In skeletal muscle, these cysteine residues mediate the regulation of SERCA activity by reversible oxidation through peroxynitrite-induced glutathionylation, resulting in increased SERCA activity; however, excessive pathological oxidative stress will cause irreversible oxidation of cysteines including sulfonylation, causing reduced SERCA activity and impaired ability to regulate through glutathionylation [1]. Our group also reported previously that SERCA activity was impaired in skeletal muscle in a mouse model of high oxidative stress in response to a lack of CuZnSOD (*Sod1*KO). This is a genetically modified mouse model that exhibits a number of accelerated aging phenotypes including muscle atrophy and weakness [54]. Based on that study, we measured the SERCA activity in naturally aged muscles (26 months old mouse model) recently and also found evidence of increased oxidative stress, including increased ROS production along with dramatically impaired SERCA activity (41% loss) [55]. In response to oxidative inactivation of SERCA function, the calcium regulation in skeletal muscles may be impaired resulting in an elevated cytosolic Ca^2+^ concentration. This in turn has been reported to be detrimental to the excitation-contraction coupling (E-C coupling) system, as this system highly relies on the stable Ca^2+^ homeostasis balancing the Ca^2+^ release from ryanodine receptors (RyRs) during the contraction and the re-uptaking by SERCA during the relaxation [3, 7]. A defect in the EC coupling system will in turn compromise muscle functions, for example the decreased force generation, prolonged time to reach the peak force with the increased half relaxation time, and damaged the key calcium handling proteins in EC coupling pathway, i.e., ryanodine receptor, and all of these can eventually contribute to a final muscle weakness and sarcopenia [53]. In addition to affecting the EC coupling system, stable Ca^2+^ homeostasis is also important for mitochondria as Ca^2+^ signaling and the calcium concentration in mitochondria is critical for mitochondrial respiratory function [67]. For example, during ATP production, Ca^2+^ ions are required for activating the ATP synthase complex [66]; moreover, the activity of some dehydrogenases in the tricarboxylic acid cycle (TCA cycle) is also regulated by Ca^2+^ ions [20]. In physiological conditions, cytosolic Ca^2+^ signals are rapidly transduced into the mitochondrial matrix, although the uptake machinery in mitochondria has a lower affinity to Ca^2+^ relative to the SR [8]. Thus, an elevated cytosolic Ca^2+^ concentration caused by SERCA dysfunction can lead to altered mitochondrial function, negatively impacting cellular energy metabolism and even leading to cell death [8]. Mitochondria in a high cytosolic calcium environment in turn may produce more ROS, exacerbating the negative effect of oxidative stress on SERCA activity and contractile dysfunction in skeletal muscles, as we reported previously [2]. The potential associations between SERCA pumps and some critical cellular events in the face of an elevated oxidative stress are illustrated in Fig. 3. Because all of these events are closely associated with muscle metabolism and functions, dysfunctional SERCA pumps can influence muscle functions through a number of pathways.

### The effects of neurodegenerative disorders and muscle disease on SERCA function

Neurodegenerative disorders such as amyotrophic lateral sclerosis (ALS) alter neuromuscular transmission through the disruption of neuromuscular junction (NMJ) structures and function and are associated with a number of denervation phenotypes in skeletal muscles [35]. The NMJ has been shown to be a highly age-related structure with its morphology of the pre- and post-synaptic regions altered and the number of neurotransmitter-containing synaptic vesicles decreased [24, 59]. The effects of NMJ dysfunction on skeletal muscles have been well reported, and SERCA activity has been reported to be dramatically reduced in denervated muscles. This could be due to the oxidative stress initiated by mitochondrial dysfunction and LOOH generation in response to denervation [28]; however, their exact interactions and underlying mechanisms are still not fully understood. Using the ALS mouse model (G93A), our group found that the loss of muscle innervation in this model was associated with a decrease in SERCA activity (~ 20% decrease in muscle from G93A mice compared to wildtype mice), although the SERCA protein level was not reduced (This unpublished data was provided by Rizwan Qaisar & Holly Van Remmen). In addition, in a recent study, we showed that overexpression of catalase targeted to skeletal muscle mitochondria (mMCAT) in the *Sod1*KO mouse muscle atrophy model led to reduced oxidative stress in muscle associated with full rescue of oxidative stress induced alterations in NMJ morphology and function, and SERCA activity is also returned to wildtype levels [70]. These studies support the close association between SERCA activity and muscle innervation status.

Other than neurodegenerative disorders, muscular dystrophies (MDs) are also a group of degenerative muscle disorders characterized by progressive muscle wasting and often premature death. Commonly, the primary defect to most MDs involves disruption of the dystrophin-glycoprotein complex (DGC), which leads to sarcolemma instability and an abnormal Ca^2+^ influx, inducing cellular necrosis [16, 19]. Duchenne muscular dystrophy (DMD) is a severe form of MD that has been studied extensively using the dystrophic *mdx* mouse model. In this model, SERCA activity is decreased more than 20% compared to wild type muscle in both hindlimb and diaphragm muscles. The impaired SERCA function contributes to the calcium dysregulation in dystrophic muscles [18], and enhancing the SERCA activity, i.e., by overexpression SERCA protein, suppresses the muscle degenerative phenotypes [19, 41]. Together, these studies suggest an important role of SERCA function under the diseased status of skeletal muscle.

### SERCA activity in muscle in response to denervation

Currently, there are two potential factors that have been studied regarding the influence of denervation on SERCA function, one is through phospholamban (PLN) [28], and the other is via receptor activator nuclear factor-kB (RANK) [14, 15]. As mentioned above, PLN is an inhibitor of SERCA pumps expressing in all muscle types. The data from Komatsu’s study show that with denervation, the amount of PLN is elevated in all major fiber types, and as much as 3-fold in MHCIIx fibers [28]. Along with the increase of PLN, the SERCA activity, mainly in fast-twitch muscle, and the calcium content in the SR are found to be reduced, strongly suggesting that in this case of denervation, PLN is a major contributor to the inhibition of SERCA activity. It is noteworthy that the elevation of PLN in this study is due to a post-translational effect, i.e., they did not find any change in the mRNA level of PLN in skeletal muscles, suggesting that some potential post-translational pathways exert modifications on PLN. However, the post-translational regulation of PLN in skeletal muscle is still elusive, although some reports that have indicated PLN is degraded by proteasome or autophagy pathways in a ubiquitin-dependent manner in cardiac muscle [45, 65, 71]. Thus, it is possible that ubiquitin-dependent modifications play a role in PLN modification in skeletal muscle as well. It is possible that denervation of skeletal muscles might suppress ubiquitination of PLN by a mechanism such as down-regulation of E3 ubiquitin ligase specific to PLN leading to an increase in the amount of PLN [28].

Receptor-activator of nuclear factor-κB (RANK), its ligand RANKL, and the soluble decoy receptor osteoprotegerin (OPG) are members of the tumor necrosis factor superfamily that regulate bone remodeling [29, 32]. The study of the regulating roles of Ca^2+^ through the interactions between RANK and RANKL is still a newly emerging area, but its function related to regulating cellular Ca^2+^ storage and a series of Ca^2+^ pumps in denervated muscles has been measured [14, 15]. In a muscle-specific RANK knock out mouse model, they demonstrated that in denervated fast twitch muscles (EDL), the RANK deletion markedly increased the content of STIM 1, a Ca^2+^ sensor, the SERCA activity, the SR Ca^2+^ storage, and also the corresponding muscular functions, such as force production and muscle fatigability [15]. This study established a novel role of RANK signaling in regulating Ca^2+^ homeostasis in skeletal muscles in responding to denervation. While this study is still preliminary and the long-term effects remain to be determined, these results open a new field of research and new therapeutic avenues for neuromuscular disease in aging.

## SERCA pumps as a potential therapeutic target

### The effect of SERCA activation on skeletal muscle functions and neurodegenerative disorders

As discussed above, SERCA pumps are implicated in many diseases, and as a result targeting SERCA as a potential therapeutic strategy has attracted significant attention in recent years. Currently, there are two primary approaches for targeting the role of SERCA, one is to increase SERCA activity through stimulating SERCA by SERCA activators [57], and the other is to control SERCA expression through genetic manipulations [37].

There are a series of mechanisms that have been shown to activate SERCA, including glutathionylation [1, 34], SUMOylation [13, 22], acetylation [42], and allosteric activation [31], and a number of corresponding drugs have been well tested (Table. 2). For example, in skeletal muscle, our group recently tested the effects of a novel allosteric SERCA activator, CDN1163, on impaired muscle functions induced by high oxidative stress [54]. CDN1163 was firstly identified in a screen by the Kang group in an insulin resistance and type 2 diabetic model (ob/ob mice) [26]. By treating the ob/ob mice with CDN1163, along with the elevated SERCA activity, they found a number of beneficial alterations in metabolisms in these mice, including reduced adipose tissue, increased energy expenditure, attenuated ER stress response and ER stress-induced apoptosis, and improved mitochondrial biogenesis [26]. In our study, we tested the effectiveness of CDN1163 as an intervention to restoring the impaired SERCA function, and we reported in muscle of the CuZnSOD knock out (*Sod1*KO) mice. From our results, we found that CDN1163 is effective for restoring SERCA activity in *Sod1*KO mice without altering SERCA protein expression and that the restoration of SERCA activity prevented muscle atrophy in *Sod1*KO mice and in addition the contractile dysfunction is dramatically improved. Elevated SERCA activity in the muscle of the *Sod1*KO mice also was associated with reduced mitochondrial ROS generation suggesting an indirect beneficial effect of mitochondrial function [54]. In addition, our group further applied this activator, CDN1163, in aged mice (26 months old); similarly, it efficiently activated SERCA function and prevented muscle from atrophy and weakness at such an old age. In addition to these positive alterations on muscle, we also found beneficial changes in gene expression, for example, activation of AMPK signaling pathways for mitochondrial restoration and maintaining muscle mass, inhibition p38 MAPK pathway for reducing stress response, and also an upregulation of calcium related genes in responding to calcium signaling and handling. In summary, muscle atrophy and weakness are always coupled with reduced SERCA activity, and a defected SERCA function will then lead to increased ER/SR stress, which will in turn impair mitochondrial functions. Hence, as a newly emerging and promising intervention, restoration of SERCA activity by activators is efficient to improve muscle functions and metabolism and further mitigate sarcopenic phenotype.

**Table 2:** Established mechanisms and tested drugs for stimulating SERCA via post-translational modifications or protein to protein interactions. The table lists the proved typical SERCA stimulating interactions, including glutathionylation, SUMOylation, acetylation and the activator through allosteric effect, and their corresponding regulating sites and effective drugs

SERCA stimulating interactions	Regulating sites	Effective drugs	Reference
Glutathionylation	Disulfide bond formed at cysteine residue of SERCA	Nitroxyl (HNO) Nitric oxide (ONOO)	Adachi et al. [1], Lancel et al. [34]
SUMOylation	SUMO binds to lysine residue of SERCA	Luteolin	Du et al. [13], Hu et al. [22]
Acetylation	Addition of acetyl group at lysine residue on SERCA	Suberanilohydroxamic acid (HDAC inhibitor)	Meraviglia et al. [42]
Allosteric activator		CDN1163	Krajnak and Dahl [31], Qaisar et al. [54]

The studies described above outline the effects of aging and muscle pathologies on SERCA in muscle and the potential for interventions that activate SERCA. In addition, the effects of neuronal SERCA stimulation on improving neurodegenerative disorders has also been investigated [57]. For instance, Alzheimer’s disease (AD) is the most common neurodegenerative disease in elderly patients, and neuronal loss with the accumulation of β-amyloid peptide are the main features in AD neural tissues [23]. Amyloid precursor protein (APP) is a critical protein in the mechanisms underlying AD, and the Aβ fragments generated by the APP protein are the main constituents of the amyloid plaques in AD neural tissues [68]. It has been shown that the regulation of Aβ production can be altered by changes in the homeostasis of the endoplasmic reticulum Ca^2+^ pool being a key point in this process [10, 27]. Parisiadou et al. have reported that the stimulation of neuronal SERCA can increase the ER Ca^2+^ store followed by the less influx of extracellular calcium to the cytosol of neuro cells, which in turn increases the interactions between APP and homer proteins, and hence reduces the accumulation of the amyloid plaques and further ameliorate the symptoms of AD [50].

### Genetic manipulations of SERCA in disease intervention

The genetic manipulation of SERCA has been extensively studied using transgenic animals, but predominantly in cardiac tissue and with respect to heart function. The major strategy for genetically manipulating SERCA pumps has been to overexpress the specific SERCA isoforms (i.e., SERCA 2a) in certain tissues (i.e., cardiac muscle) to rebalance the calcium homeostasis [43, 51]. Many studies used adenoviral (AAV-1) SERCA 2a as a gene tool to increase the expression of the SERCA 2a gene in animals or in human ventricular myocytes. Universally, these studies found that the overexpression of SERCA 2a isoforms efficiently increased the SERCA activity in cardiac tissues, and enhanced the contraction and relaxation velocity, and hence restored contractile function to normal levels [11, 21]. More recently, using AAV-1 mediated SERCA 2a gene transfer, it was found that the electrophysiological and mechanical functions were improved in the hearts from myocardial infarction (MI) models [43]. As for the skeletal muscle specific isoform SERCA 1, a popular way to overexpress SERCA 1 is to use a modified human skeletal muscle α-actinin promotor to selectively increase its amount in fast-twitch muscles as described by Goonasekera et al. [19]. Some previous studies have indicated that the increased SERCA 1 expression in skeletal muscles improves calcium handling and muscle function and reduces markers of muscular damages in some muscular dystrophy mouse models, such as the mdx mice and the δ-sarcoglycan–null (*Sgcd*^*–/–*^) mice [19, 41]. Overall, all of these findings suggest that restoring Ca^2+^ transport by increasing SERCA levels via overexpression is effective for maintaining contractility and further improving the mechanical functions of muscles.

## Conclusions

The SERCA pump plays a critical role in Ca^2+^ regulating. The proper function of SERCA is important in skeletal muscle metabolism. Substantial data has demonstrated that restoration or activation of SERCA activity through either stimulating the SERCA pumps or genetically manipulating the SERCA expressions may be an effective therapeutic approach for the treatment of some diseases, such as sarcopenia, cardiomyopathy, and some neurodegenerative disorders such as AD. Future studies targeting the underlying mechanisms of SERCA activity and its role in tissue metabolism and associated pathways are needed to further reveal a deep understanding of SERCA functions in various diseases conditions.

## Data Availability

Data sharing is not applicable to this review, as no datasets were generated or analyzed in this article.

## Abbreviations

SERCA: Sarcoendoplasmic reticulum calcium ATPase
SR: Sarcoendoplasmic reticulum
MD: Muscle dystrophy
PMCA: Plasma membrane Ca^2+^ ATPase
E-C coupling: Excitation-contraction coupling
RyR: Ryanodine receptor
MMPs: Matrix metalloproteinases
CICR: Calcium-induced calcium release
PLN: Phospholamban
SLN: Sarcolipin
DWORF: Dwarf open reading frame
MLN: Myoregulin
*Sod1*KO: Copper zinc superoxide dismutase knock out
TCA cycle: Tricarboxylic acid cycle
ROS: Reactive oxygen species
ALS: Amyotrophic lateral sclerosis
NMJ: Neuromuscular junction
mMCAT: Skeletal muscle-specific mitochondrial catalase
DGC: Dystrophin-glycoprotein complex
DMD: Duchenne muscular dystrophy
RANK: Receptor activator nuclear factor-kB
OPG: Osteoprotegerin
EDL: Extensor digitorum longus
AD: Alzheimer’s disease
APP: Amyloid precursor protein
AAV-1: Adenoviral
Sgcd^-/-^: δ-sarcoglycan–null
MI: Myocardial infarction
